# Effects of Pre-Exercise High and Low Glycaemic Meal on Intermittent Sprint and Endurance Exercise Performance

**DOI:** 10.3390/sports7080188

**Published:** 2019-08-05

**Authors:** Man Tong Chua, Govindasamy Balasekaran, Mohammed Ihsan, Abdul Rashid Aziz

**Affiliations:** 1 Physical Education and Sports Science Academic Group, National Institute of Education, Nanyang Technological University, Singapore 637616, Singapore; 2 Athlete Health and Performance Centre, Aspetar Orthopaedic and Sport Medicine Hospital, Doha 29222, Qatar; 3 Sport Science and Medicine, Singapore Sport Institute, Sport Singapore, Singapore 397630, Singapore

**Keywords:** pre-ingestion, carbohydrate, glucose response, fasting, heart rate, ratings of perceived exertion

## Abstract

The purpose of this study is to investigate the effects of ingesting either a high glycaemic index (HGI) or low glycaemic index (LGI) carbohydrate meal (preceding a 12 h overnight fast and where the meal was ingested 45-min prior to activity) on intermittent sprint and endurance exercise performance. Ten male varsity athletes from intermittent sports (age 23.6 ± 1.7 years, VO_2max_ 51.9 ± 4.7 mL·kg^−1^·min^−1^) underwent a peak velocity (V_peak_) test and familiarisation session, followed by two experimental sessions in random order. Experimental sessions involved the ingestion of either an HGI or LGI meal, followed by the completion of the modified Loughborough Intermittent Shuttle Test (mLIST). There was no significant difference between HGI or LGI meals on sprint times (*p* = 0.62) and distance to exhaustion (*p* = 0.54) in the mLIST. Exercise heart rate, blood lactate and ratings of perceived exertion were also similar between the two meal trials throughout the mLIST (all *p* > 0.05). Subjective ratings of hunger, fullness, satiety and satisfaction were also not significantly different between the two meals. In conclusion, consuming either an HGI or LGI meal after a prolonged 12 h fast and ingesting the meal 45 min prior to exercise did not differ in either physiological, subjective and intermittent sprint and endurance performance outcomes.

## 1. Introduction

The glycaemic index (GI) was established to enable the comparison of human physiological responses towards varying types of food and to develop food items which, when ingested, would lead to a relatively slower or more gradual increase in blood glucose concentration levels, especially for people with diabetes or glucose intolerance [1]. Calculation of the GI value of the food is based on an individual’s postprandial glycaemia response after the ingestion of a CHO-containing food in comparison to the response observed from the consumption of an equivalent portion of reference food (i.e., 50 g of glucose) [1]. In this regard, high glycaemic index (HGI) foods (GI value of >70), are rapidly digested and absorbed into the body, resulting in rapid increases in blood glucose levels per unit of CHO in comparison to low glycaemic index (LGI) foods (GI value of <55), which are digested and absorbed in a more gradual fashion, leading to a relatively smaller rise in blood glucose concentration levels [2,3].

Interestingly, the knowledge of the GI of different foods has since been used to examine the influence of these varying foods on substrate metabolism during exercise. A plethora of research has been conducted to investigate the effects of ingesting LGI and HGI food within 60 min to between 1 to 3 h prior to exercise performance, especially in endurance exercise performance using time-trials and time-to-exhaustion exercise protocols (see reviews) [4,5,6]. Although there are controversies, the latest review [6] indicated, in general, there were significant improvements in exercise capacity and/or endurance performance outcome following the ingestion of LGI foods as compared to moderate of high GI foods. The common mechanism for the improved exercise performance in these studies was attributed to a greater fat oxidation (and therefore preserving the endogenous glycogen stores) because of lower insulin response after the ingestion of an LGI meal (where insulin has been known to inhibit fat oxidation). The glycogen-sparing effects will hence result in a greater availability and thus, oxidation of CHO throughout the exercise. This, the authors argued, would led to a higher, sustained energy production towards the end of the exercise, thus improving exercise performance [7]. 

There have been only very few studies that examined the effects of consuming HGI and LGI food prior to intermittent-type exercise performance [8,9,10,11,12]. Instead of exercising at a constant steady-state submaximal intensity, the activity of intermittent exercise is usually made-up of repeated bouts of maximal or near-maximal exercise efforts (typically a few seconds in duration) interspersed with relatively longer durations of moderate or low-intensity recovery periods. During the work periods, anaerobic-derived substrates (i.e., phosphocreatine and glycolysis from the breakdown of intramuscular CHO) provide the fuel required for the rapid breakdown of adenosine triphosphates for muscular contractions; and with the aerobic or oxidative systems, primarily from either endogenous CHO and fats sources, helping to supply the energy substrates to restore muscular homoeostasis such as the replenishment of tissue oxygen stores, the resynthesis of phosphocreatine, and the metabolism of accumulated lactate during the recovery periods. Low GI, as compared to HGI foods, has been shown to promote the use of fats throughout the duration of a prolonged exercise [10,11,12]. It may be reasoned that ingesting LGI foods prior to an intermittent exercise protocol may perhaps, promote greater fat oxidation during the recovery periods (and therefore enhance the recovery capabilities and sparing of the body’s limited glycogen stores during these periods). Indeed, Goto [9] showed improved sprint performance during the Loughborough Intermittent Shuttle Test (LIST) in well-trained female soccer players who had consumed LGI food 3 h prior to the exercise [9]. 

It is common for athletes, or even among the general population, to commence exercise after being in a fasted state for a prolonged period of 10–12 h. For example, many athletes prefer to exercise very early in the morning. Hence, upon waking after an overnight fast, they may subsequently consume a small, light meal just before the commencement of early morning exercise. Another good example is in the month of Ramadan, many Muslim athletes would observe their religious fasting during the daylight hours which typically last between 10–16 h duration, hence their sporting activities may be postponed until after the breaking of the day’s fast [13]; in this case, the fasted athletes would consume their *iftar* meal (i.e., breaking of day’s fast) before commencing their exercise a short time later. Therefore, the purpose of this study is to compare the effects of ingesting either an HGI or LGI meal (preceding a 12 h overnight fast and the meal is consumed ~45 min prior to exercise) on intermittent sprint and endurance exercise performance. Based on previous studies [7,9,14,15], it is hypothesised that a pre-exercise LGI meal would elicit superior results as compared to a pre-exercise HGI meal.

## 2. Methods

### 2.1. Subjects

Ten healthy male varsity athletes from intermittent sports (age 23.6 ± 1.7 years; stature 174.1 ± 5.7 cm; body mass 70.9 ± 6.5 kg; peak running velocity or V_peak_ 16 ± 0.7 km·h^−1^; maximal oxygen consumption or VO_2max_ 51.9 ± 4.7 mL·kg^−1^·min^−1^) volunteered for the study. Participants were involved in their respective intermittent sport training sessions but were asked to rest completely 24 h before each experimental session. Participants were amateur-level athletes who were training between 6 to 8 sessions per week, which consisted of sport-specific technical skills, aerobic and anaerobic (speed) conditioning as well as resistance training. 

### 2.2. Experimental Design

The present study adopted a single-blind, controlled, randomized, cross-over trial experimental design. Participants made a total of three visits to the laboratory, between 3 to 7 days apart. The first visit was a preliminary test and familiarisation session. The preliminary test consisted of the V_peak_ test and familiarisation of the modified Loughborough Intermittent Shuttle Test (mLIST). The second and third visits were the two experimental sessions, i.e., either the HGI or LGI meal trials. These two sessions were randomised across the tested individuals comparing the participants’ subjective and physiological responses, and intermittent sprint and endurance exercise performance.

### 2.3. Test Meals

The HGI meal was glutinous chicken-flavoured rice, marketed locally as Lor Mai Kai (Kong Guan Food Private Limited, Singapore). The LGI meal provided was wholemeal bread with coconut jam and butter spread, known locally as Kaya Butter Toast. The LGI was physically prepared by the primary investigator to match the caloric and macronutrient profile of the Lor Mai Kai. Both the LGI and HGI meals were of the same caloric values of ~425 kcal (Table 1), equivalent to between ~0.9 and 1.1 g CHO·kg^−1^ body mass. The GI values of the LGI and HGI meal in the present study were determined based on the food items reported in the study of Sun et al. [16]. The study examined the GI meals of selected popular local foods of Singapore (note: The present study was conducted in Singapore). In the study of Sun et al. [16] which was conducted on 47 individuals from the same population cohort as that of the present study, the GI values of the same food items that were used in the present study were directly determined using the standardized glucose response method [16].

### 2.4. Experimental Protocol

Participants were not informed of the true aim of the study. They were duly informed that the rationale of this research was to test two types of pre-exercise meals commonly found in Singapore on intermittent exercise performance, i.e., bread vs. rice. This helped ensure that the true intention of the study was concealed, thus minimising potential placebo effects [17]. Participants were, however, debriefed at the end of the study, and at the same time the true purpose of the study and the results of their performance tests were then made known.

Participants underwent two experimental sessions, separated by a ~3–7 day period. They were instructed to abstain from strenuous physical activity 24 h prior to each experimental trial and abstain from alcohol, caffeine and any ergogenic supplements. To limit any influence of nutritional and physical activity habits on exercise performance, subjects kept a 24 h diet and physical activity record on the day before the first experimental trial. They were instructed to follow the same dietary and physical activity pattern for a 24 h period before the second experimental session. All trials were also scheduled at the same time of the day to avoid any circadian rhythm influence on exercise metabolism and exercise performance. Participants were instructed to cease any ingestion of food and liquid (except water) the evening (by 22:00 h) before the day of the exercise until their reporting time the next morning (by 10:00 h). This is to ensure that they reported to the laboratory in a 12 h fasted state. Upon arrival to the laboratory, participants ingested either an LGI meal or HGI meal together with 500 mL of plain water within 10–15 min. Participants then remain rested for 30 min before the commencement of the warm-up to begin the intermittent sprint and endurance performance test.

Before the experimental trials, participants completed a familiarisation session during which all the experimental procedures were taken and questionnaires were explained to them. Participants then completed the V_peak_ test to determine their individual running velocities for the modified Loughborough Intermittent Shuttle Test (mLIST) exercise protocol. The V_peak_ test was conducted on a motorised treadmill, which commenced at 8.0 km·h^−1^; at a gradient of 1% for 1 min, after which the speed was increased by 1.0 km·h^−1^; in 1 min increments until volitional exhaustion was attained [18]. The individual’s V_peak_ and maximum heart rate (HR_max_) was the highest treadmill velocity maintained for 60-s and the highest 5-s average data, respectively. During the V_peak_ test, the individual’s VO_2max_ was also measured with indirect calorimetry using a calibrated metabolic cart (TrueOne 2400, ParvoMedics, Salt Lake City, UT, USA). Measurements of heart rate (HR; H10, Polar Electro Oy, Kempele, Finland) and ratings of perceived exertion (RPE 6–20 scale) [19] were monitored throughout the test. After the V_peak_ test, participants rested for 15 min and this was followed by a 15 min familiarisation trial of the mLIST.

### 2.5. Intermittent Sprint and Endurance Performance Test

**Figure 1:**
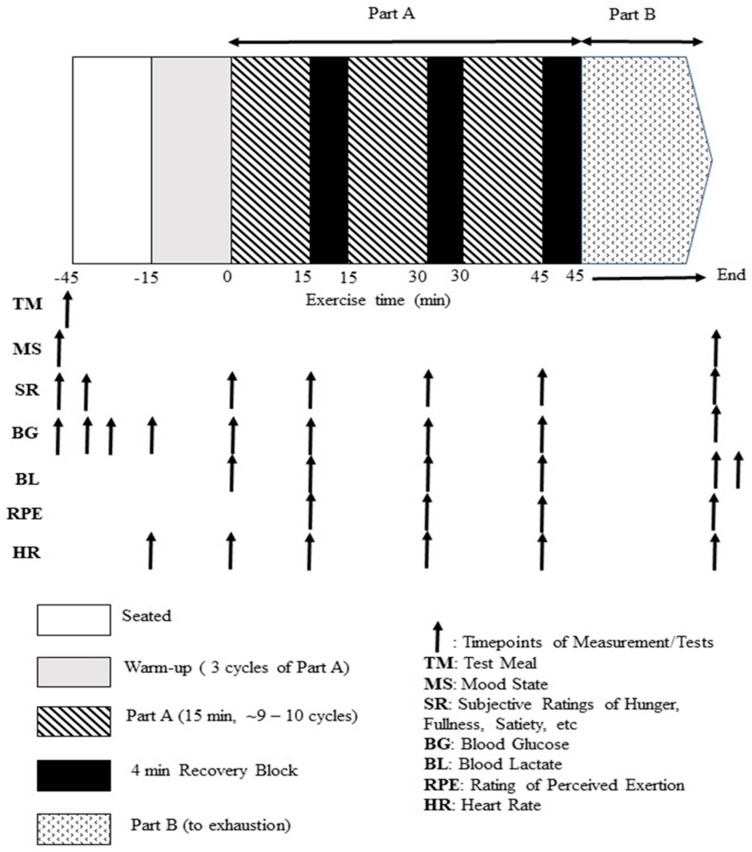
Schematic representation of the experimental design of the study.

The present study used a modification of the original LIST [20], i.e., mLIST (Figure 1). The test requires the participant to run between two lines, 20 m apart, at varying submaximal and sprinting speeds for 45 min (3 × 15 min exercise blocks) with 4 min passive recovery between blocks (i.e., part A of mLIST), followed by shuttle runs until volitional exhaustion (i.e., part B of mLIST), for a total exercise duration of between 50 to 70 min. Individuals’ exercise intensities for jogging and running were based on percentages of V_peak_ determined from the V_peak_ test, except for the 15 m maximal sprint effort. Each of the 15 min exercise blocks of part A consisted of ~9–10 cycles of 3 × 20 m walk (at 1.3 m·s^−1^), followed by 1 × 15 m maximal sprint, 3 × 20 m jog (at 55% V_peak_) and 3 × 20 m run (at 95% V_peak_). Part B consisted of 20 m shuttles alternating between running speeds corresponding to 55% and 95% V_peak_ until volitional exhaustion (defined as the inability to maintain pace for three consecutive shuttles at 95% V_peak_). During the test, participants were instructed to follow the pattern of movements (i.e., to perform the walk, jog and fast running in accordance to the speed dictated by the audible sounds), except for the sprinting component where the player sprinted the 15 m distance as fast as possible i.e., all-out maximal effort for every sprint. In the mLIST, sprints were initiated from a standing position, 20 cm behind the start timing gate. Sprint times were recorded with the Speed Light Sports Timing system (Swift Performance Equipment, Lismore, NSW, Australia) to an accuracy of 1/100th of a second. The light gates were at a standardised height of 1.2 m above the ground, placed at the start and at the 15 m mark. The coefficient of variation for part A and part B in the original LIST was estimated to be ~5% and ~30% (based on duration to exhaustion), respectively [20]. The mean sprint times in part A and distance to exhaustion in part B of the mLIST were taken as criterion measures of intermittent sprint and endurance exercise performance, respectively. No other food except fluid (only plain water was allowed ad libitum) was allowed during the mLIST. All exercise sessions were conducted on a running track inside an air-conditioned indoor gymnasium. The ambient temperature and relative humidity throughout these sessions were between 21 and 23 °C and between 55 and 65%.

### 2.6. Physiological and Subjective Measures

Heart rate via short-range telemetry (H10, Polar Electro Oy, Kempele, Finland) was taken throughout exercise. Capillary blood samples to assess blood glucose concentration via finger-prick were taken before consumption of the test meal, 5-, 15-, 30- and 45-min after ingestion of the test meal, using a portable glucose meter (Accu-chek Performa, Roche Diagnostics GmbH, Mannheim, Germany). Capillary blood samples were additionally taken at the end of each block of exercise in part A and upon completion of part B of the mLIST to assess blood lactate concentration (Lactate Pro 2, Arkray Inc., Kyoto, Japan). The players rated their subjective RPE using Borg’s categorical 6–20 scale at pre-exercise and at the end of each exercise block of part A and at the end of part B of the mLIST.

Before and at the end the mLIST, participants completed the Brunel University Mood State (BRUMS). The BRUMS comprised of 32 items of descriptors for six different mood subscales of anger, confusion, depression, fatigue, tension and vigour [21]. Raw scores for each mood subscale were totalled and used for statistical analysis. Participants were also instructed to rate their subjective ratings of hunger, satisfaction, fullness and satiety on a 100 mm visual analogue scale with descriptor words located at each end to express the two most extreme ratings [22]. These questions were administered before consumption of the test meal, then at 5 min, 45 min after ingestion, during recovery periods of part A and after completion of part B of the mLIST.

### 2.7. Statistical Analysis

All data were analysed using statistical software (IBM SPSS Version 24, Chicago, IL, USA). Shapiro-Wilks was conducted to assess the normality of the data. Sprint times; blood glucose; blood lactate; HR; RPE; subjective ratings of hunger, satisfaction, fullness and satiety; and the various mood states were all analysed by two-factor trial x time repeated measures of analysis of variance (ANOVAs). Mauchly’s test was consulted and Green-Geisser correction was applied if sphericity was violated. If a significant main effect was observed, post-hoc paired sample *t*-tests with Bonferroni adjustments were used to detect the occurrences of the differences. Paired sample *t*-test was employed to compare the variable distance to exhaustion between the two meal conditions. Significance level was set at *p <* 0.05. Supplementary analysis using magnitude-based differences were also undertaken for all performance variables (i.e., sprint times and distance to exhaustion) [23]. Changes were analysed in raw units (90% confidence intervals) relative to the smallest worthwhile change (SWC; 0.2× between subject SD) determined from pooled sprint (block 1) and distance to exhaustion data. Chances of an increase or decrease were evaluated qualitatively as <1%, almost certainly not; 1–5%, very unlikely; 25–75%, possible; 75–95%, likely; 95–99%, very likely; >99% almost certainly. Additionally, changes were considered unsubstantial/unclear if the probability of an increase and decrease were both >5% [24].

## 3. Results

### 3.1. Exercise Performance

Table 2 depicts mean sprint times for each block of exercise, the overall mean sprint times and the distance to exhaustion, in the two test meal trials. A significant time effect on sprint times was observed in both meal conditions (*p* = 0.05), but no significant difference was found between the two meal trials at any time point throughout the mLIST (*p* > 0.05, η_p_^2^ = 0.33). There was no interaction effect between trial X time for mean sprint times across three exercise blocks (*p* = 0.62, η_p_^2^ = 0.37). For the distance to exhaustion performance, paired sample *t*-test showed no significant difference in the distance covered between the two meal trials (*p* = 0.54), although the distance to exhaustion in the HGI trial was 82 ± 28 m greater (difference of ~7.7 ± 2.7% with a small effect size, 0.23) than in the LGI trial. The qualitative outcome across the sprints and distance to exhaustion were both deemed as unclear.

### 3.2. Blood Glucose and Blood Lactate Concentration

**Figure 2:**
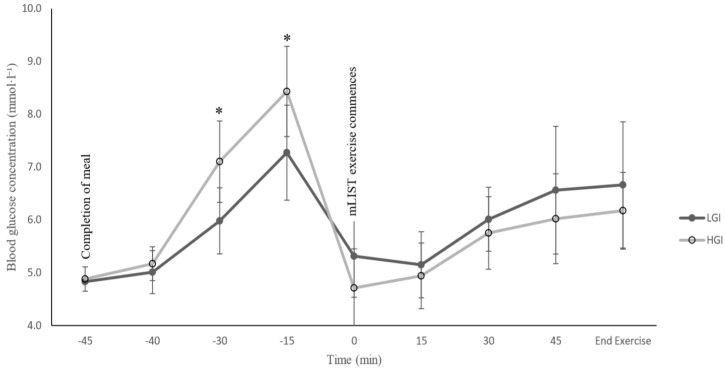
Measurements of blood glucose concentration over the duration of the experimental session. LGI = low glycaemic index; HGI = and high glycaemic index; * *p <* 0.01, significantly different between LGI and HGI meal trials

**Figure 3:**
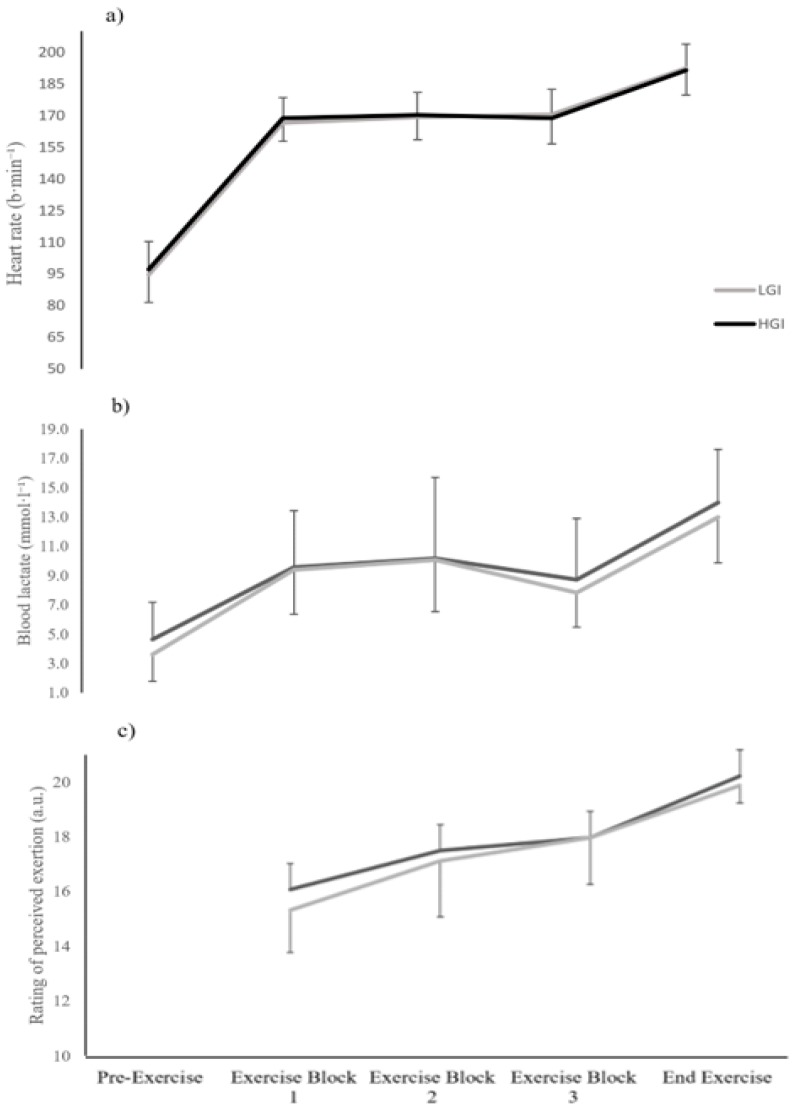
Heart rate (**a**), blood lactate (**b**) and ratings of perceived exertion (**c**) measures in the low glycaemic index (LGI) and high glycaemic index (HGI) meal trials during the modified Loughborough Intermittent Shuttle Test exercise.

A significant trial X time interaction effect was found (*p* = 0.002, η_p_^2^ = 0.46) in blood glucose concentration during the post-prandial non-exercise period. Post-hoc test showed a significant difference in blood glucose concentration between HGI and LGI trials after post-meal ingestion at the 15- and 30-min mark (*p* = 0.007 and 0.003, respectively; Figure 2). A statistically significant main effect for time was observed (*p* = 0.000, η_p_^2^ = 0.60) in blood glucose concentration throughout the mLIST exercise, but no significant interaction effect of trial X time was found (*p* = 0.68, η_p_^2^ = 0.05). Similarly, blood lactate concentration showed a progressive increase due to exercise (all *p* values <0.05; Figure 3). However, there were no significant differences between the two meal trials at each time point throughout the mLIST (all *p* values > 0.05).

### 3.3. Heart Rate and Ratings of Perceived Exertion

There were significant main effects for time factors for both HR and RPE (both *p* < 0.01, η_p_^2^ = 0.98 and 0.85, respectively; Figure 3). Both variables were progressively increasing with the duration of exercise. More importantly, there were no significant interaction effects of trial X time for both HR and RPE during exercise (*p* = 0.43 and 0.54, η_p_^2^ = 0.09 and 0.07, respectively). 

### 3.4. Subjective Ratings of Hunger, Satisfaction, Fullness and Satiety and Mood States

**Figure 4:**
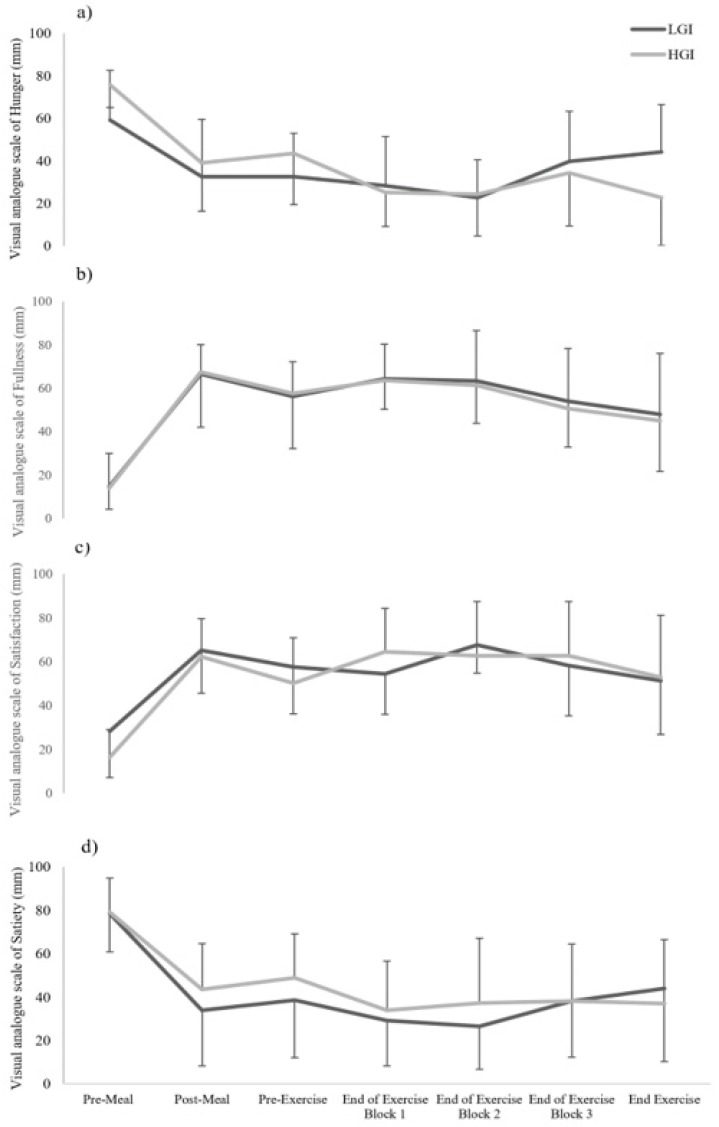
Subjective ratings of hunger (**a**), fullness (**b**), satisfaction (**c**) and satiety (**d**) in the low glycaemic index (LGI) and high glycaemic index (HGI) meal trials at pre- and post-meal ingestion and throughout the exercise duration.

There were significant main effects for time for all ratings of hunger, satisfaction, fullness and satiety (all *p* values < 0.01; Figure 4). There were no significant trial X time interaction effects for the subjective ratings of hunger, satisfaction, fullness and satiety (*p* = 0.50, 0.16, 0.79, and 0.36, η_p_^2^ = 0.26, 0.17, 0.05, and 0.11, respectively). Similarly, there were significant main effects on time for fatigue, and tension subscales (both *p* values < 0.01; Table 3). More importantly, there were no significant differences between the LGI and HGI meal trials in all these subjective measures (all *p* > 0.05), except for the mood subscale of vigour, (*p* < 0.05; Table 3). An interesting finding in the present study was the significant increase in levels of vigour, from pre- to post-mLIST in the HGI meal trial (from 5.3 ± 1.9 to 9.6 ± 3.5 mm, *p* = 0.002), but not in the LGI meal trial (from 7.2 ± 3.0 to 7.8 ± 4.5 mm, *p* = 0.69).

## 4. Discussion

The aim of this study was to examine the effects of pre-exercise HGI and LGI meals (preceding a 12 h overnight fast and where the meal was ingested 45 min prior to activity) on intermittent sprint and endurance exercise performance. The results indicated that there were no significant differences between LGI and HGI meal trials for sprint times (part A) and distance to exhaustion (part B) of the mLIST, and thus, contrary to our initial hypothesis, ingestion of a pre-exercise LGI meal relative to a HGI meal did not lead to any ergogenic effects on intermittent sprint and endurance performance.

The present study finding is supported by previous studies that have shown no differences in exercise performance capacity between either ingesting LGI and HGI meals on intermittent exercise performance [8,9,10,11,12]. However, it should be noted that the intermittent protocols used in these four studies were somewhat different from that of the present study. For example, sprint performance consisting of five 60 s sprints was assessed only during the last 15 min of a 90 min high-intensity intermittent running [8,9,10,11] and during a 1-kilometre time-trial after 90 min of intermittent exercise [12]. Therefore, differences in the manner of the sprints performed as well as in the total duration of the exercise have made the comparison between the results of these four studies with that of the present study difficult. 

With regards to intermittent exercise performance protocol that is similar to the present study, only one study [9] has shown the ergogenic effects of ingesting an LGI meal relative to HGI meal. The study’s author showed that sprinting during the original LIST exercise protocol was faster with a concomitant lower HR and RPE in the LGI meal trial relative to the HGI meal trial [9]. The potential mechanism mentioned for the enhanced sprint performance during the LGI meal trial in that study was due to a higher blood glucose concentration in the LGI meal trial relative to the HGI meal trial, at the start and end of the LIST exercise. However, it should be mentioned that this view was a mere speculation by the author since the subjects’ blood glucose levels in the study of Goto [9] were not actually measured. Interestingly, in the present study, blood glucose concentration was shown to be relatively higher throughout the mLIST exercise in the LGI meal trial compared to the HGI meal trial, albeit the difference was not statistically significant (Figure 2); nonetheless, the higher blood glucose level did not seem to translate to any real sprint and endurance performance enhancement in the LGI trial (Table 2). Additionally, the present study also showed no statistically significant differences in blood lactate concentration, HR and RPE between the LGI and HGI meal trial. We have no explanation for the contrasting findings of the Goto [9] study versus the present study. However, there are several stark differences in the methods of the two studies, including differences in the pre-ingestion time prior to the exercise (present study: 45 min vs. Goto: 3 h, before the commencement of the exercise), the total duration of the exercise (present study: ~60 min vs. Goto: ~90 min), the amount of food ingested pre-exercise (present study: ~0.9–1.1 vs. Goto: ~2.0 CHO·kg^−1^ body mass) and the participants’ characteristics (present study: males and relatively not as well-trained as Goto’s subjects vs. Goto: females and well-trained soccer players). 

During prolonged moderate- to high-intensity continuous exercise protocols, there is an increased reliance on free fatty acid oxidation and economical utilisation and sparing of limited endogenous glycogen stores, which may accrue an advantage in consuming an LGI vs. HGI meal during exercise [7,14,15]. However, in the present study, the dynamic intermittent nature of mLIST involves moderate- to very-high-intensity exercise (i.e., work) interspersed with recovery of low intensity. For such a pattern of exercise, a high percentage of energy used during work is supplied through anaerobic energy metabolism whilst aerobic metabolism is primarily responsible for lower-intensity recovery efforts like walk, jog and passive rest (for the purpose of re-synthesising phosphocreatine and other homeostatic processes) [25]. Therefore, the overall energy metabolism of the present study’s intermittent exercise protocol suggests a much greater involvement of anaerobic metabolism during the mLIST exercise, i.e., a heavily diminished usage of free fatty acid oxidation, which could possibly lead to the inability to maximise the full benefits of consuming the LGI meal [8,12]. 

Negative mood state is usually associated with low blood glucose concentration levels [26]. Thus, an interesting finding in the present study was the significantly enhanced vigour levels in the HGI meal trial, concomitant with much lower blood glucose levels throughout mLIST exercise relative to the LGI meal trial (Figure 2). Although participants were not aware of the true intention of the research, the form of the two experimental meals may have caused an unintentional placebo effect [17]. It is well-established that rice is a staple food in the local diet (where the study is conducted), especially in the form of long-grained rice [27]. Thus, participants may have felt that ingesting the HGI meal (which is rice-based) provided them with a relatively higher amount of ‘energy’ as compared to ingesting the LGI meal (which is bread-based). The ingestion of the former may have empowered the individuals with a greater perceived sense of physical vigour, especially late during exercise, i.e., during part B of the mLIST.

There are several limitations of the present study which should be highlighted. This research study involved a small number of participants, albeit similar to that previously of many other GI related studies [10,12]. As such, the lack of statistical significance in several key variables, for example, the distance to exhaustion, could be because the study is slightly underpowered. Participants in the present study were university-level athletes from intermittent sports, which implied that the current findings may not be directly translated to elite-level athletes. Substrate oxidation to assess the involvement of free fatty acid and CHO oxidation during the mLIST was not directly measured, but perhaps in hindsight, this could have been done with a portable indirect calorimetry gas analyser during exercise, which could help to provide a clearer relationship in the metabolic preference after consumption of the two test meals. 

## 5. Conclusions

This study compared the effects of pre-exercise HGI and LGI meals (preceding a 12 h overnight fast and where the meal was ingested 45 min prior to activity) on an intermittent sprint and endurance exercise protocol. Results showed that ingesting either HGI or LGI meals did not significantly influence intermittent sprint and endurance performance or any other physiological and subjective variables measured. Thus, intermittent sport athletes may ingest either an LGI or HGI meal if they are planning to engage in exercise within an hour post-prandial.

## Figures and Tables

**Table 1:** Nutritional information of the two test meals.

Food Item	High Glycaemic Index Meal (Lo Mai Kai)	Low Glycaemic Index Meal (Kaya Butter Toast)
Serving size (g)	179	115
Energy (kcal)	~425	~424
Carbohydrates (g)	70	70
Protein (g)	13.1	12
Fat (g)	10.3	10.3
Glycaemic index *	~106	~49
Ingredients	Glutinous rice, chicken, sesame oil, sugar, oyster sauce (sugar, salt, monosodium glutamate, oyster extract (oyster, salt), modified corn starch, wheat flour, yeast extract, caramel, sorbic acid), salt, palm olein, soya sauce (soybean, salt, sugar, wheat flour), monosodium glutamate, pepper.	Soft wholemeal bread (wholemeal wheat flour with bran and wheat germ, wheat flour (unbleached), wheat gluten, honey, wheat bran, skimmed milk powder, common salt, vegetable oil (palm), dextrose, Baker’s yeast, yeast nutrients (ammonium sulphate, sodium chloride, calcium sulphate) emulsifiers, thiamine, riboflavin, niacin, iron, calcium propionate). Olive spread (rapeseed oil, olive oil, sunflower oil, palm fraction, water, skimmed milk powder, salt and vitamins, emulsifiers, stabilizer, preservative, flavouring and colouring substances). Kaya (consists of sugar, egg, coconut milk, modified tapioca starch, pandan juice).

* Based on the glyceamic index values of similar food items in Sun et al. [16].

**Table 2:** Results of exercise performance measures in the two test meal trials.

Performance Variables	LGI Meal Trial	HGI Meal Trial	*Mean Difference (CI)*	*Percentage Benefit/Trivial/Harmful*	*Qualitative Outcome*
Mean sprint time in exercise block 1 (s)	2.54 ± 0.08	2.54 ± 0.09	0.01 (−0.06–0.07)	36/38/27	Unclear
Mean sprint time in exercise block 2 (s)	2.55 ± 0.11	2.56 ± 0.08	0.01 (−0.07–0.08)	39/35/26	Unclear
Mean sprint time in exercise block 3 (s)	2.59 ± 0.14	2.57 ± 0.07	0.02 (−0.0–0.07)	24/29/47	Unclear
Overall mean sprint time in part A of mLIST (s)	2.56 ± 0.11	2.56 ± 0.08	0.001 (−0.07–0.07)	31/37/32	Unclear
Distance to exhaustion in part B of mLIST (s)	1024 ± 381	1106 ± 409	82 (−200–365)	50/30/17	Unclear

LGI = low glycaemic index; HGI = high glycaemic index; mLIST = modified Loughborough Intermittent Shuttle Test; CI = confidence interval.

**Table 3:** Mood subscales prior to the meal ingestion of the experimental meal and after the completion of the exercise.

	LGI Meal Trial		HGI Meal Trial		*Test of Within Subjects’ Effect*		
Mood state	At Pre-ingestion	Post-mLIST exercise	At Pre-ingestion	Post-mLIST exercise	*Time (p-value)*	*Trial (p-value)*	*Time X Trial (p-value)*
Anger	1.5 ± 2.6	1.6 ± 3.4	0.6 ± 1.4	0.3 ± 0.5	0.63	0.20	0.64
Calmness	8.4 ± 3.2	7.4 ± 4.1	8.4 ± 2.3	7.8 ± 2.4	0.47	0.79	0.76
Confusion	0.3 ± 0.5	0.6 ± 1.1	0.6 ± 1.2	0.6 ± 0.9	0.66	0.62	0.4
Depression	0.8 ± 2.1	0.8 ± 2.1	0.3 ± 0.7	0.3 ± 0.7	1.0	0.47	1.0
Fatigue	4.4 ± 2.3	9.8 ± 3.0	4.0 ± 1.8	8.8 ± 2.7	0.000 *	0.53	0.63
Happiness	6.8 ± 3.5	8.1 ± 5.1	7.3 ± 2.3	9.0 ± 3.4	0.16	0.40	0.84
Tension	2.1 ± 2.5	0.1 ± 0.4	1.1 ± 1.6	0.1 ± 0.4	0.048 *	0.07	0.07
Vigour	7.3 ± 3.4	7.3 ± 4.2	5.4 ± 1.5	9.4 ± 3.8	0.14	0.89	0.025 *

* Scores were based on a minimum and maximum score of 0 and 16. mLIST = modified Loughborough Intermittent Shuttle Test; LGI = low glycaemic index; HGI = high glycaemic index; * *p* < 0.05 statistically significant.
